# Efficacy of fibrin glue versus sutures for attaching conjunctival autografts in pterygium surgery: a systematic review with meta-analysis and trial sequential analysis of evidence

**DOI:** 10.18632/oncotarget.17195

**Published:** 2017-04-18

**Authors:** Aihua Lan, Feifan Xiao, Yun Wang, Zhen Luo, Qixin Cao

**Affiliations:** ^1^ First Clinical Academy, Guangxi Medical University, Nanning, Guangxi, China; ^2^ Department of Ophthalmology, Ningbo Eye Hospital, Ningbo, Zhejiang, China; ^3^ Department of Ophthalmology, Liuzhou Worker's Hospital, Liuzhou, Guangxi, China; ^4^ Department of Ophthalmology, Huzhou Hospital of Traditional Chinese Medicine, Huzhou, Zhejiang, China

**Keywords:** fibrin glue, suture, pterygium, meta-analysis, trial sequential analysis

## Abstract

Previous meta-analyses have been conducted to compare the efficacy of fibrin glue (FG) versus sutures in pterygium surgery; however, additional clinical trials have since been published. Therefore, we conducted an updated meta-analysis to further explore the association between FG application in pterygium surgery, and the recurrence rate, complication rate, and surgical duration. An electronic literature search for eligible studies published before July 29, 2016 was conducted across multiple databases. Odds ratios (ORs), standardized mean difference (SMD), and 95% confidence intervals (CI) were calculated. Publication bias of the included articles was evaluated by funnel plots. Differences in recurrence rate and complication rate between the FG and suture groups were evaluated in terms of OR with 95% CI, and SMD with 95% CI were used to estimate the difference in surgical duration. Trial sequential analysis (TSA) was used to determine whether the currently available evidence was sufficient and conclusive. Twenty-four studies were included in this study. The pooled ORs for recurrence rate and complication rate were 0.35 and 1.121, respectively. The pooled SMD for surgical duration was −4.142. The TSA results indicated that evidence of the effect was sufficient in the recurrence group and surgical duration group. Although there was no difference in complication rate between FG and sutures, the apparent advantages of FG over sutures are shorter surgical duration and greater reduction in the recurrence rate of pterygium.

## INTRODUCTION

Pterygium is a common ocular surface growth disorder originating in the conjunctiva and extending to the cornea [1]; its incidence ranges from 0.7% to 31% [2, 3]. The standard treatment for pterygium is surgical excision, but the recurrence rate after surgery can be as high as 24%–89%, which compromises outcomes [4]. Farid et al. [5] reported that the average time to recurrence was 3.13 months. Based on the simple excision of pterygium, multiple strategies and techniques have been developed to reduce the high rate of pterygium recurrence, including limbal conjunctival autograft [6], human amniotic membrane grafting [7], beta-irradiation [8], stem cell transplantation [9], mitomycin-C [10] and fibrin glue (FG) [11, 12]. However, severe complications might be induced by pterygium surgery, such as autograft dehiscence and corneal lesions [13, 14]. Conjunctival or limbal conjunctival autograft are suggested to be the best treatment with a low recurrence rate ranging from 1.9% to 5.3%, and high safety according to some studies [15–18]. Furthermore, they have been demonstrated to be more effective at treating recurrent pterygium than other methods [19].

Traditionally, the most common method of conjunctival autograft fixation in pterygium surgery is the use of absorbable or non-absorbable sutures. However, this prolongs surgical duration and is associated with several complications, such as sub-conjunctival hemorrhage, proliferative granuloma, and corneal epitheliopathy [20, 21]. In 2004, Koranyi et al. [22] reported that the use of FG instead of sutures when attaching the conjunctival transplant in pterygium surgery causes significantly less postoperative pain and significantly shortens surgical duration. Therefore, FG as an alternative to sutures for conjunctival autograft fixation, has been applied in pterygium surgery.

FG contains equal amounts of two components: aprotinin, along with a thrombin CaCl_2_ solution, and fibrinogen mixed with factor XIII [23]. Although many clinical trials have compared the efficacy of FG versus sutures in pterygium surgery, the results have not always been consistent. Moreover, the conclusion of a single clinical trial is limited by its relatively small sample size, shorter duration of follow-up, and difference in patient populations and surgical technique compared with other studies. Furthermore, the extent to which differences in the study region, patients and inconsistencies in case and control inclusion criteria between studies explain the conflicting outcomes is unknown. Therefore, meta-analysis provides a useful tool for the measurement of heterogeneity [24]. In fact, two published meta-analyses compared the efficacy of FG versus sutures in pterygium surgery in 2011. Pan et al. [25] included seven RCTs [13, 22, 23, 26–29] involving 342 participants with 366 eyes, and Shi et al. [30] included 366 eyes from seven RCTs [13, 22, 26–29, 31]. Additionally, Shi et al. [30] did not assess the quality of the included studies. Furthermore, only the recurrence rate, complication rate, and surgical duration were measured in these previous meta-analyses, stratified analyses including region, sample size, duration of follow-up, and suture material were not conducted.

Additional clinical trials have since been conducted to compare the efficacy of FG versus sutures in pterygium surgery. Therefore, we performed an updated meta-analysis to further explore the association between the application of FG in pterygium surgery and the recurrence rate, complication rate, and surgical duration. A greater number clinical trials with larger sample sizes were included in our meta-analysis, and several stratified analyses including region, study type, suture material, sample size, follow-up time, and study quality score were conducted.

## RESULTS

### Literature search and characteristics of selected studies

**Figure 1:**
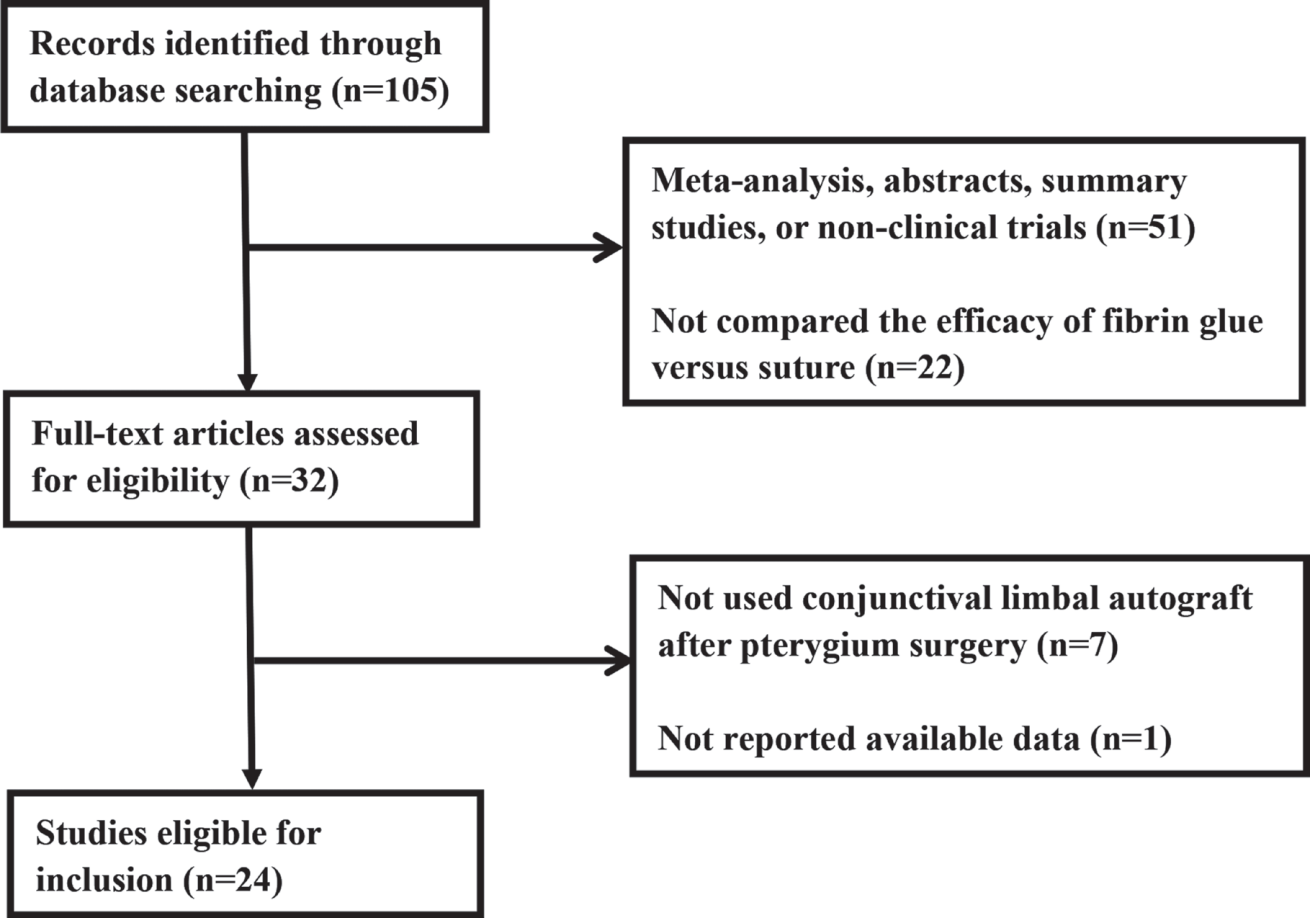
Flow chart showing the selection process for the included studies

Initially, 105 relevant studies were obtained through a literature search. We excluded 51 studies after a review of the abstracts because they were meta-analyses, abstracts, summary studies, or not clinical trials, and 22 studies were excluded because they did not compare between FG versus suture. We then conducted a full-text review of the remaining 32 articles. Seven studies were excluded because these did not use conjunctival autograft in pterygium surgery, and one due to lack of available data [3]. Therefore, 24 clinical trials [5, 13, 14, 20–23, 26–29, 31–43] comparing the efficacy of FG versus sutures for limbal conjunctival autograft fixation in pterygium surgery were selected for inclusion in the meta-analysis. The flow chart of candidate study selection is summarized in Figure 1.

A total of 1839 eyes in 24 included clinical trials published from 2004 to 2015 were enrolled in this meta-analysis. Among the 24 clinical trials, 16 studies were randomized controlled trials (RCTs), and the remaining eight trials were non-RCTs, including retrospective studies and simple comparative studies. Seventeen of the studies included Asian participants and seven included non-Asian participants. The sample sizes of these clinical trials ranged from 17 to 461, and the mean patient age ranged from 40.1 to 63 years. The sutures applied in the suture group included vicryl and nylon (i.e., absorbable and non-absorbable sutures). The duration of follow-up ranged from 2 to 29 months. Jadad score was applied to assess the quality of the included RCTs; nine RCTs obtained a score of at least 3 indicating high-quality studies, while seven RCTs scored 2 indicating low-quality studies. The characteristics and quality score of the selected clinical trials are summarized in Table 1.

**Table 1:** The characteristics and quality score of the selected clinical trials

Author	Year	Country	Study type	Type of suture	Age (FG/SE)	Sample size		Recurrence (%)		Complications (%)		Operating time (FG/SE)				Follow-up (month)	Jadad score
						FG	SE	FG	SE	FG	SE	Mean	SD	Mean	SD		
Koranyi G [22]	2004	Sweden	RCT	Vicryl 7-0	44/48	20	23	2(10%)	4(18%)	0(0%)	0(0%)	9.70	2.48	18.50	6.6	6.00	3
Uy HS [13]	2005	Philippines	RCT	nylon 10-0	45/45	11	11	0(0%)	0(0%)	1(10%)	1(10%)	27.80	1.00	67.00	3.60	12.00	5
Jiang J [23]	2008	China	RCT	nylon 10–0	57/57	20	20	1(5%)	2(10%)	3(15%)	1(5%)	17.90	2.60	31.80	3.3	6.00	2
Ozdamar Y [28]	2008	Turkey	RCT	Vicryl 8-0	42.6/42.6	12	12	0(0%)	0(0%)	0(0%)	0(0%)	/	/	/	/	12.00	4
Karalezli A [27]	2008	Turkey	RCT	Vicryl 8-0	53.4/58.5	25	25	1(4%)	3(12%)	7(28%)	12(48%)	15.70	2.40	32.50	6.70	12.00	3
Farid M [5]	2009	USA	RCT	Vicryl 8-0	47.3/52.2	27	20	1(3.7%)	4(20%)	/	/	/	/	/	/	12.00	3
Coral-Ghanem R [33]	2010	Brazil	RCT	Vicryl 8-0	42.6/49.3	106	58	12(11.32%)	15(26%)	/	/	/	/	/	/	12.00	3
Malik VK [34]	2010	India	RCT	Vicryl 8-0	60.08/60.36	25	25	1(4%)	3(12%)	2(8%)	7(28%)	18.24	2.20	26.60	1.93	13.60	2
Cagatay HH [21]	2014	Turkey	RCT	Vicryl 8-0	52.08/53.75	53	53	1(1.89%)	4(8%)	5(10%)	7(14%)	/	/	/	/	6.00	3
Vichare N [37]	2013	India	RCT	nylon 10–0	46.8/49.5	30	30	1(3.33%)	3(10%)	/	/	34.43	4.94	50.93	4.96	13.00	3
Sati A [38]	2014	India	RCT	Vicryl 8-0	40.1/40.9	30	30	2(6.67%)	3(10%)	2(7%)	0(0%)	15.50	1.20	27.63	1.63	6.00	3
Cui B [31]	2009	China	RCT	nylon 10-0	52/55	20	20	0(0%)	0(0%)	0(0%)	0(0%)	20.50	3.60	42.80	4.5	6.00	2
Lei QF [41]	2015	China	RCT	nylon 10-0	52/50	30	30	1(3.33%)	3(10%)	/	/	24.50	6.50	35.20	5.4	6.00	2
Hu JF [43]	2012	China	RCT	Vicryl 8-0	55.45/53.42	46	43	2(4.35%)	6(14%)	/	/	30.65	9.43	46.56	10.24	24.00	2
Hu QM [42]	2014	China	RCT	nylon 10-0	52.14/53.14	40	36	/	/	/	/	18.33	0.50	39.13	1.02	9.00	2
Liu H [40]	2013	China	RCT	nylon 10-0	/	30	30	0(0%)	0(0%)	1(4%)	0(0%)	20.32	17.35	28.79	19.53	6.00	2
Koranyi G [14]	2005	Sweden	non RCT	Vicryl	44/48	258	123	14(5.43%)	17(14%)	13(6%)	11(9%)	/	/	/	/	2.00	/
Miranda-Rollón MD [32]	2009	Spanish	non RCT	Vicryl 8-0	59.8/59.8	8	9	1(12.5%)	0(0%)	1(13%)	4(45%)	/	/	/	/	29.00	/
Hall RC [26]	2009	New Zealand	non RCT	Vicryl 8-0	47.8/49.8	24	23	0(0%)	2(9%)	10(42%)	0(0%)	12.04	3.03	26.04	5.00	23.00	/
Ratnalingam V [29]	2010	Malaysia	non RCT	Vicryl 8-0	60.7/60.7	68	69	3(4.41%)	11(16%)	0(0%)	0(0%)	16.93	2.85	29.84	5.65	1.00	/
Rubin MR [35]	2011	Brazil	non RCT	nylon 10-0	45.6/47.8	21	26	1(4.76%)	2(8%)	1(5%)	1(4%)	19.05	6.12	48.15	7.13	6.00	/
Yüksel B [20]	2010	Turkey	non RCT	Vicryl 8-0	53.6/54.6	29	29	2(6.9%)	4(14%)	2(7%)	0(0%)	23.42	13.34	41.45	3.2	21.50	/
Cha DM [36]	2012	Korea	non RCT	nylon 10–0	63.0/56.9	22	30	1(4.55%)	6(20%)	18(82%)	16(54%)	27.71	5.22	43.30	8.18	6.00	/
Küçükerdönmez C [39]	2014	Turkey	non RCT	Vicryl 8-0	57.1/52.1	13	13	0(0%)	0(0%)	/	/	/	/	/	/	5.00	/

/: not available; FG: fibrin glue group; SE: suture group; SD: standard deviation.

### Recurrence rate, complication rate, and surgical duration

**Figure 2:**
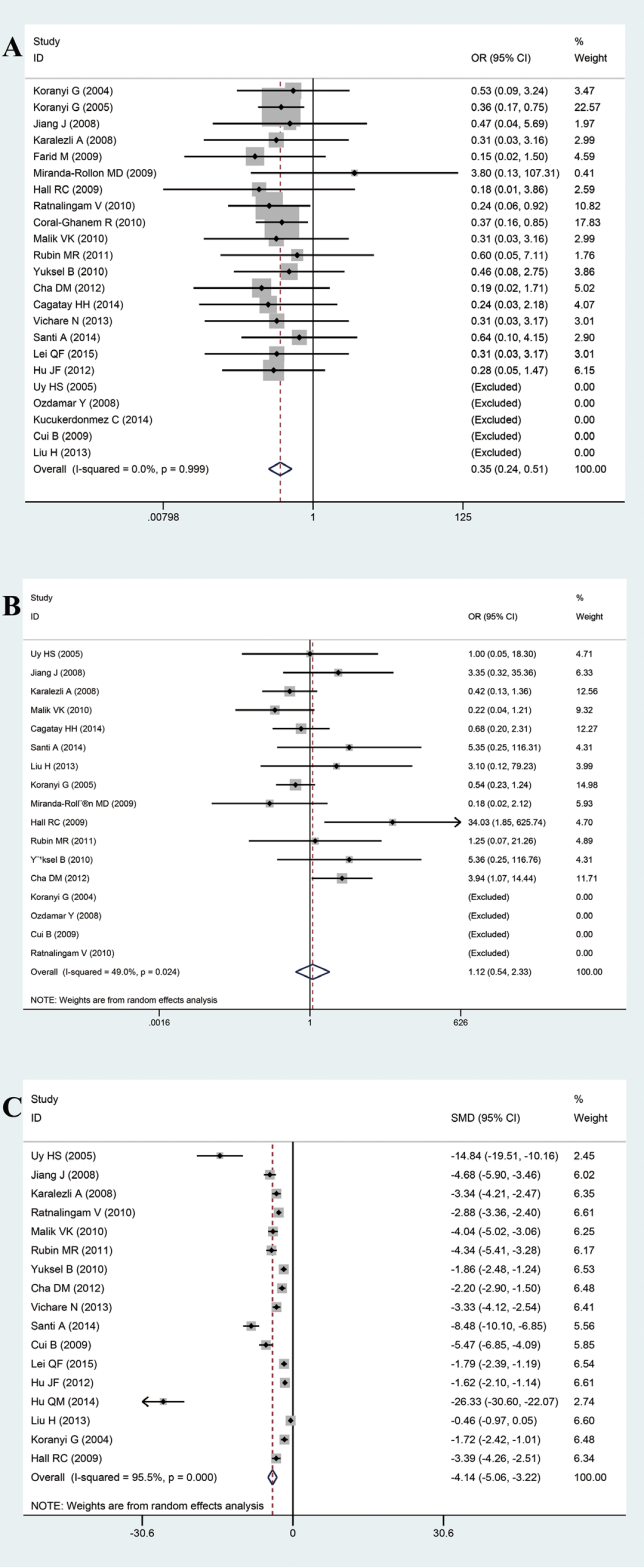
(**A**) Forest plot of the ORs for the effect of FG versus sutures on the combined outcome “recurrence rate”. (**B**) Forest plot of the ORs for the effect of FG versus sutures on the combined outcome “complication rate”. (**C**) Forest plot for the effect of FG versus sutures on the combined outcome “surgical duration”.

Twenty-three included studies reported the recurrence rate, 17 studies reported the complication rate, and 17 studies compared the surgical duration between FG and suture groups. The pooled odd ratios (ORs) for the recurrence rate and complication rate were 0.35 (95% confidence interval [CI]: 0.24–0.51, *P <* 0.001, I^2^ = 0.0%, Figure 2A) under a fixed-effects model and 1.121 (95% CI: 0.540–2.326, *P* = 0.759, I^2^ = 49.0%, Figure 2B) under a random-effects model, respectively. The results showed that FG was more effective at reducing the recurrence rate and did not increase the complication rate. The pooled standardized mean difference (SMD) for surgical duration was −4.142 (95% CI: −5.06–3.22, *P <* 0.001, I^2^ = 95.5%, Figure 2C) under the random-effects model. The surgical duration in the FG group was significantly reduced compared with the suture group.

### Stratified analyses

In order to further explore whether FG was associated with the recurrence rate and complication rate, we conducted stratified analysis of recurrence rate and complication rate based on region (Asia and non-Asia), study type (RCT and non-RCT), suture material (vicryl and nylon), sample size (> 50 eyes and ≤ 50 eyes), follow-up duration (> 6 months and ≤6 months) and study quality score (≥ 3 and ≤ 2 in RCTs). The ORs are summarized in Table 2. The stratified analyses did not significantly change the complication rate and recurrence rate results, with the exception that in studies with a quality score of 2 (OR = 3.987, 95% CI: 1.297–12.255, *P* = 0.016, I^2^ = 0.0%), FG significantly increased the complication rate, while in studies with at least a 6 month follow-up duration (OR = 0.49, 95% CI: 0.25–0.98, *P* = 0.043, I^2^ = 0.0%), FG significantly reduced the complication rate. Complications including hemorrhage, dehiscence, inflammation, corneal lesions, and retraction were selected to conduct stratified analyses. The ORs are shown in Table 2; there was no statistical significance in the sub-group analyses of complication rate, with the exception that the retraction group suggested that FG was associated with an increased retraction rate under the fixed-effects model (OR = 6.398, 95% CI: 1.629–25.125, *P* = 0.008, I^2^ = 0.0%). Due to the lack of studies with a RCT quality score of 2 (*n* = 3), studies with at a least 6 month follow-up duration (*n* = 6), and retraction studies (*n* = 4), further research should be conducted to confirm these findings.

**Table 2:** Summarized ORs or SMD in this meta-analysis

Group			Study	Sample size (eye)	OR	95% CI	*P*	I^2^ (%)	Statistical model
Main outcomes	Recurrence rate		23	1680	0.348	(0.239~0.506)	< 0.001*	0.00	Fixed-effects model
	Complication rate		17	1234	1.121	(0.540~2.326)	0.759	49.00	Random-effects model
	Surgery time		17	991	−4.142^a^	(−5.060~-3.224)	< 0.001*	95.50	Random-effects model
Recurrence rate/ Complication rate	Region	Asia	16/12	934/604	0.311/1.257	(0.173~0.559)/(0.417~3.786 )	< 0.001*/0.684	0/57.9	Fixed/Random
		non-Asia	7/5	746/630	0.38/0.822	(0.233~0.619)/(0.366-1.849)	< 0.001*/0.636	0/20.8	Fixed/Random
								
	Study type	RCT	15/10	915/543	0.344/1.088	(0.205~0.578)/(0.385~3.072)	< 0.001*/0.874	0/45.6	Fixed/Random
		non-RCT	8/7	765/691	0.351/1.270	(0.204~0.604)/(0.385~4.187)	< 0.001*/0.695	0/60.5	Fixed/Random
								
	Suture material	Vicryl	15/11	1299/650	0.351/2.561	(0.235~0.525)/(0.556~11.791)	< 0.001*/0.227	0/70.5	Fixed/Random
		Nylon	8/6	381/584	0.326/0.642	(0.115~0.920)/(0.316~1.305)	0.034*/0.221	0/11.2	Fixed/Random
								
	Sample size	> 50 eyes	11/7	1227/384	0.403/2.492	(0.180~0.903)/(0.581~10.695)	0.027*/0.059	0/51.5	Fixed/Random
		≤ 50 eyes	12/10	453/854	0.333/0.602	(0.218~0.508)/(0.356~1.019 )	< 0.001*/0.219	0/0	Fixed/Random
								
	Follow-up	> 6 months	11/6	628/365	0.349/0.49	(0.199~0.612)/(0.25~0.98)	< 0.001*/0.043	0/0	Fixed/Random
		≤ 6 months	12/11	1052/869	0.346/2.61	(0.209~0.573)/(0.80~8.48)	< 0.001*/0.027	0/57.9	Fixed/Random
								
	Quality score	≥ 3 scores	9/6	576/349	0.353/0.426	(0.195~0.641)/(0.178~1.018)	0.001*/0.055	0/0	Fixed/Random
		≤ 2 scores	6/4	339/194	0.319/3.987	(0.111~0.916)/(1.297~12.255)	0.034*/0.016*	0/0	Fixed/Random
Complication	Hemorrhage		3	132	1.575	(0.532~4.658)	0.412	0.00	Fixed-effects model
	Dehiscence		9	755	1.068	(0.524~2.177)	0.857	0.00	Fixed-effects model
	Inflammation		9	801	1.102	(0.716~1.696)	0.658	33.60	Fixed-effects model
	Corneal lesions		3	537	0.543	(0.243~1.211)	0.136	0.00	Fixed-effects model
	Retraction		4	199	6.398	(1.629~25.125)	0.008*	0.00	Fixed-effects model

*: statistical significance.

^a^:SMD.

### Publication bias

**Figure 3:**
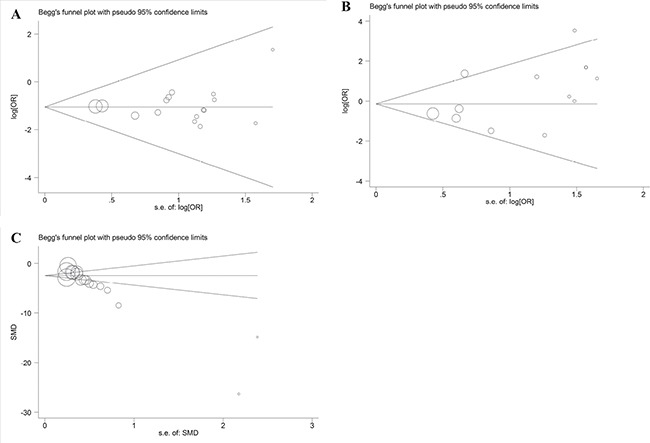
(**A**) Funnel plot for the publication bias test in the recurrence studies. (**B**) Funnel plot for the publication bias test in the complication rate analysis. (**C**) Funnel plot for the publication bias test in the surgical duration analysis.

There was no publication bias in the recurrence studies (*P* = 0.596, Figure 3A), complication rate analysis (*P* = 0.097, Figure 3B), or surgical duration analysis (*P* = 0.129, Figure 3C).

### Sensitivity analysis

To evaluate the stability of results, sensitivity analyses were performed. We removed studies one by one from the analysis; however, the result did not change with the removal of any of the studies, suggesting that our results for the recurrence (Supplementary Figure 1A), and surgical duration analysis (Supplementary Figure 1B) were consistent. Notably, in the complication rate group (Supplementary Figure 1C), after we excluded the study of Hall et al. [26], the I^2^ changed to 34.0%, but the result (OR = 0.90, 95% CI, 0.47–1.70, *P* = 0.118) did not change, suggesting that the results had sufficient statistical power.

### Meta-regression analysis

Meta-regression analysis suggested that region, sample size, suture material, and follow up duration did not have a significant impact on the heterogeneity across studies in the recurrence rate group (Supplementary Figure 2). Meta-regression with region, suture material, and follow up duration showed no significant impact on between-study heterogeneity in the complication rate group (Supplementary Figure 3). However, sample size had an impact on the heterogeneity in the complication rate group. Detailed information is shown in Supplementary Table 1.

### Trial sequential analysis

**Figure 4:**
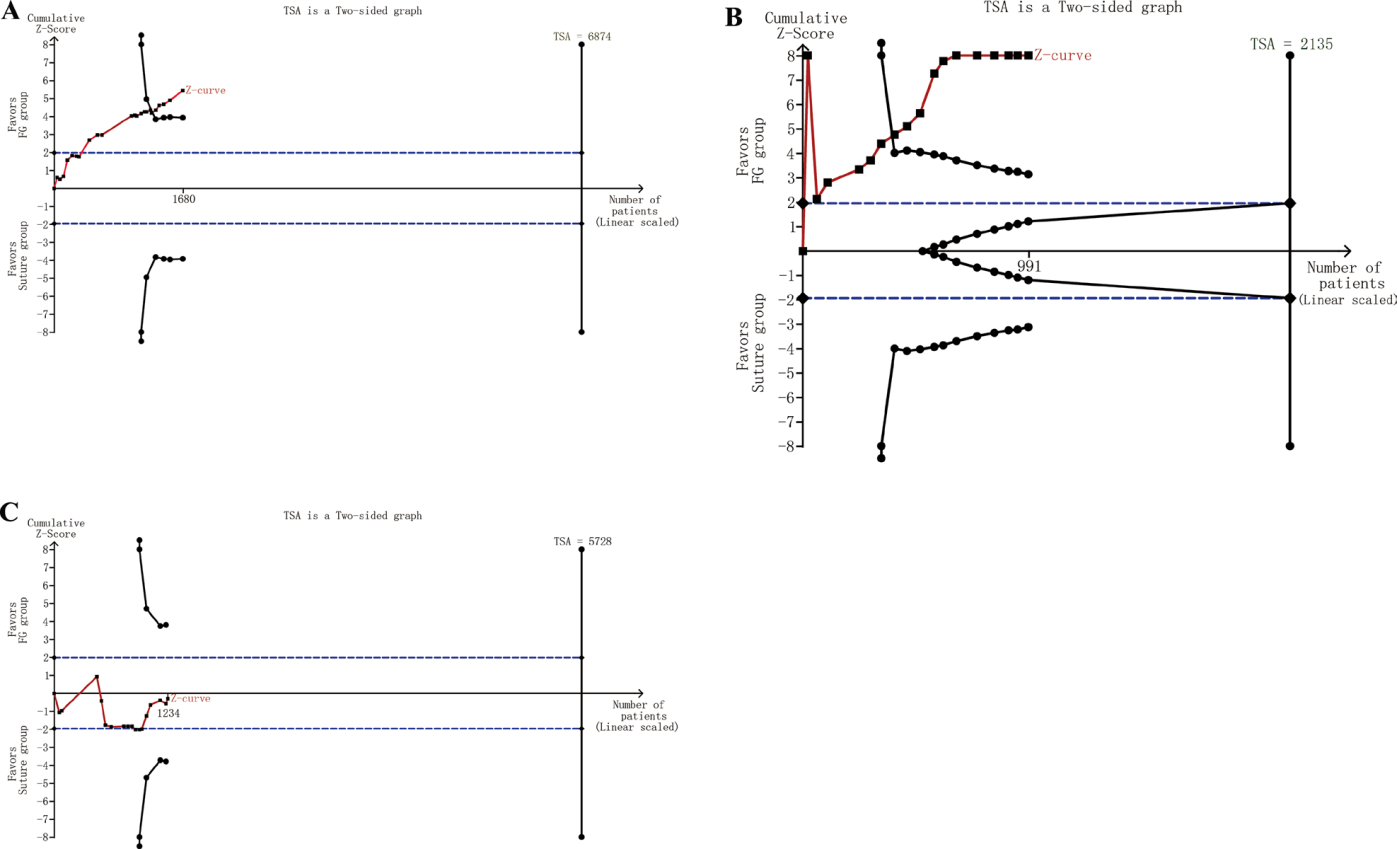
(**A**) TSA in the recurrence group. (**B**) TSA in surgical duration group. (**C**) TSA in the complication group.

Based on the theory of trial sequential analysis (TSA), in the recurrence group, the required information size to demonstrate a difference in recurrence rate between the FG and suture groups was 6,874 patients (Figure 4A). Results showed that the Z-curve crossed the trial monitoring boundary before reaching the required information size, indicating that the cumulative evidence was adequate and further trials were unnecessary. Moreover, the cumulative Z-curve crossed the trial sequential monitoring boundary, establishing sufficient and conclusive evidence in the surgical duration group (Figure 4B). However, in the complication group, the Z-curve did not cross the trial monitoring boundary before the required information size was reached (5728 patients), demonstrating that the cumulative evidence was insufficient and more studies were required to confirm this result (Figure 4C).

## DISCUSSION

We conducted an updated meta-analysis to further explore the association between the application of FG in pterygium surgery, and the recurrence rate, complication rate, and surgical duration. In total, 1,839 eyes from 24 studies were included in the meta-analysis. Based on the overall meta-analysis, we found that, compared with sutures, FG was more effective at reducing the recurrence rate, but not the complication rate. Our analysis indicated a significantly shorter surgical duration for FG compared with sutures. In the subgroup analysis, based on region, follow-up time, quality score, sample size, study type, and suture material, FG still had a lower recurrence rate. Moreover, there were no significant differences in complication rates between the two groups in terms of region, sample size, study type, and suture material. To increase the robustness of this conclusion, we performed a TSA to calculate the required information size and estimate the sample number required to make a stable conclusion.

In the 17 included studies on surgical duration, the use of FG was associated with a markedly reduced surgical time. Vichare et al. [37] showed that the mean surgical durations in the suture and FG groups were 50.93 min and 34.43 min, respectively. Mean surgical duration was also shorter in the FB group (23.42 min) than in the suture group (41.45 min) in another study by Yuksel et al. [20]. As a biological adhesive, when FG is applied to tissue, a firm and stable fibrin network is created and formed in 30 seconds, reproducing the final stage of the coagulation pathway and promoting collagen cross-linking and wound healing [44]. Furthermore, healing after the use of FB is characterized by minimal inflammation, comfortable eye, and good cosmetic outcome compared with sutures [45].

The most important issue for patients and surgeons regarding pterygium surgery is recurrence. In our meta-analysis, 23 trials reported the recurrence rate. Of these studies, five [13, 28, 31, 39, 40] reported no recurrence in both groups, which may be related to the short follow-up time. Ratnalingam et al. [29] found no significant difference between suture and FG groups at 1 month and 6 months postoperatively. However, after a 1-year follow-up, three eyes (4.41%) showed recurrence with the presence of fibrovascular tissue crossing the limbus in the 68 eyes in the FB group, which is statistically less than that in the suture group in which 11 recurrences (15.9%) were reported in the 69 eyes that underwent surgery. In our subgroup based on follow-up duration, we found that the recurrence rate in the FG group was lower than that in the suture group at ≥ 6 months and > 6 months. A possible reason for this difference is that our study had a more adequate sample size. However, the follow-up duration also plays an important role in recurrence time. Furthermore, the recurrence rate in the FG group was still less than that in the suture group in Asian and Non-Asian regions, indicating that FG has stable effect among different ethnicities. Moreover, the result did not change based on other subgroup analyses such as study quality score, study size, study type, and suture material.

Complication rate is another issue concerning pterygium surgery. Four studies [22, 28, 29, 31] did not report any complications in both groups. In the study by Koranyi et al. [22], complications, such as transient transplant edema and persistent corneal epithelial defects, occurred equally in both groups. Meanwhile, Jiang et al. [23] observed no significant differences in the occurrence of other complications between the two groups. Based on our 17 included studies, there was no difference in complication rate between the two groups. Subgroup analysis based on region, sample size, study type, and suture material reached the same conclusion. In the follow-up time of ≤ 6 months group, the FG group had a lower complication rate than the suture group. However, the rate of retraction was higher in the FG group. Hall et al. [26] reported retraction of the nasal edge of the graft in eight patients in the FG group, however, these cases had healed 1 month later and did not present a problem to the patient. Moreover, subgroup analysis based the quality scores in the present study showed a higher complication rate associated with FG use in studies that had a quality score of 2, which may be related to the low quality.

Significant heterogeneity was caused by a combination of different complications in the meta-analysis. Based on sensitive analysis, after we excluded the study of Hall et al. [26], I^2^ changed to 34.0%, but the result remained the same, suggesting that the analysis had sufficient statistical power. Moreover, we used RCTs to obtain more reliable results. Meta-regression indicated that sample size had an impact on the heterogeneity in the complication rate group. Based on our subgroup analysis by sample size in the complication rate group, there was high heterogeneity in the > 50 eyes group. A possible reason is that this group contained fewer studies (seven studies with 384 eyes).

Our meta-analysis has some limitations that should be acknowledged. First, the number of included studies was insufficient and the included publications were mainly written in English, and relevant studies in other languages may have been omitted. Second, not all included articles were RCTs, so more robust data with greater clinical evidence should be included in future analyses. Third, the relationship between recurrence rate and age was not analyzed due to lack of data. Fourth, the time for recurrence could not be explored due to lack of available data. Finally, only published trials with available data were analyzed in our study; therefore, the inclusion of unpublished data may influence our conclusions.

Based on the 24 included studies, our meta-analysis supports the use of FG for conjunctival autografting in pterygium surgery over sutures. Compared with sutures, FG is more effective at reducing the recurrence rate of pterygium, so patients would benefit from the use of FG. Moreover, there was no difference in complication rate between FG and sutures, indicating that FG has a similar safety compared with sutures. Furthermore, the surgical duration was significantly shorter in the FG group compared with the suture group, decreasing the burden on the medical profession. However, average surgical cost was higher when using FG [20]. In order to reduce the surgical cost, operations should be well-planned. A challenge for clinical practice is that an increasing number of patients are operated on the same day. Therefore, we recommend that doctors communicate well with patients about surgical consumables and expenses. Considering the cost, we recommend suturing for low-income patients.

In conclusion, FG is a safe and effective tool in pterygium surgery with conjunctival limbal autografting. Although there was no difference in the complication rate between FG and sutures, apparent advantages of FG are shorter surgical duration and greater reduction in pterygium recurrence rate. Based on the TSA, these conclusions of recurrence and surgical duration are robust, and further duplicated trials are unnecessary. However, more studies are required to confirm conclusion relating to complication rate.

## MATERIALS AND METHODS

### Search strategy

We conducted an electronic literature search for eligible studies published until July 29, 2016 across multiple databases, including PubMed, Web of Science, Science Direct, Cochrane Central Register of Controlled Trials, Wiley Online Library, China National Knowledge Infrastructure, China Biology Medicine Disc, Chongqing VIP, and Wan Fang Data. The keywords applied in the search were: (pterygium OR pterygia) and conjunctival autograft and suture and (adhesive fibrin tissue OR fibrin adhesive OR fibrin glue OR fibrinogen adhesive OR fibrin sealant system OR autologous fibrin tissue adhesive OR fibrin seal* OR crosseal OR fibrin klebe system immuno OR transglutine OR human fibrin sealant OR tisseel OR tissel OR tissucol OR beriplast OR FG OR tissue adhesive* OR biological glue). In addition, previous meta-analyses, review studies, and cited references from relevant articles were screened as appropriate.

### Inclusion and exclusion criteria

Two authors independently reviewed the titles and abstracts of literature to identify studies which fulfilled the inclusion criteria: (1) clinical trials; (2) patients with primary pterygium; (3) efficacy comparison between FG versus sutures; (4) pterygium surgery with conjunctival limbal autograft; and (5) availability of at least one of the following outcomes: recurrence rate, complication rate, and surgical duration. There were no language restrictions regarding the inclusion of studies. Exclusion criteria comprised the following: (1) patients with recurrent pterygium; (2) pterygium surgery with other autograft; and (3) no available data.

### Data extraction and assessment of study quality

Two authors independently assessed the quality of the selected trials and extracted the following data: first author's name, publication year, country, suture material, mean age, sample size, recurrence rate, complication rate, surgery duration, follow-up duration, and study type. The Jadad composite scale was applied to assess the quality of the included studies, which assigns scores for reported randomization, blinding and withdrawals [46]. The Jadad scale is a 5-point scale, in which a study with a score of ≥ 3 is commonly regarded as high quality and a study with a score of ≤ 2 is low quality [47]. Any disagreement between authors was resolved by discussion to reach consensus.

### Statistical analysis

For recurrence rate, we calculated the total number of recurrences at the end of follow-up in all clinical trials. For complication rate, considering the various criteria of complications in the included studies, a uniform standard was established to minimize individual variation among trials. We evaluated all complications with remarkable signs, including the displacement or loss of the autograft, dehiscence, hemorrhage, infection, corneal lesions, and other indications that required special treatment. We excluded common inflammatory reactions, such as edema, fibrosis, and proliferative granuloma, to conduct a stratified analysis. In order to achieve a more accurate analysis of complication rate, hemorrhage, dehiscence, corneal lesions, and retraction were also selected. In addition, we conducted stratified analyses on recurrence rate and complication rate based on region, study type, suture material, sample size, follow-up duration, and study quality score. For the surgical duration, we recorded the mean and standard deviation (SD) from each study. When no SD was available, and the maximum and minimum values were reported, we calculated the SD using the following formula [25]. $\sqrt{\frac{{\left(Max-Mean\right)}^{2}+{\left(Min-Mean\right)}^{2}}{4}}$ Differences in recurrence rate and complication rate between the FG and suture groups were evaluated by OR with 95% CI, and SMD with 95% CI were used to estimate the difference in surgical duration between groups. We used the I^2^-statistic to calculate heterogeneity among the studies [48], I^2^ > 50% implies significant heterogeneity resulting in the use of a random-effects model, otherwise a fixed-effects model was selected [24]. Due to the significant heterogeneity caused by a combination of different complications in the meta-analysis, we chosen a random-effects model to investigate complication rate. Publication bias was assessed using funnel plots. Meta-regression analyses assessed the influence of region, sample size, suture material, and follow up duration. All statistical analyses were performed using the STATA (version12.0, STATA Corp., College Station, TX, USA). Statistical significance was determined as *P <* 0.05.

### Trial sequential analysis

Meta-analyses may result in type I errors owing to an increased risk of random error when the studies included in the meta-analysis have a small sample size, low quality, publication bias, and whose conclusions tend to change by later studies with larger sample sizes [49]. TSA not only allows for controlling the *P-value* when scarce data exist and clear conclusions cannot be drawn, but also allows the quantification of the required sample size for determining the effect whilst adjusting the threshold for statistical significance [50]. Therefore, the required information size was based on the assumption of a plausible relative risk of 30% with low risk bias, and we adopted the risks for a type I error (α) of 5%, and a type II error (β) of 20%. TSA software version 0.9 beta (http://www.ctu.dk/tsa) was used in this study. If a TSA monitoring boundary is crossed with the Z-curve before reaching the required information size, robust evidence can be confirmed and further reduplicative studies are unnecessary. Otherwise, it is necessary to continue performing trials [51].

## SUPPLEMENTARY MATERIALS FIGURES AND TABLES


