# Supplementary material for: Efficacy of fibrin glue versus sutures for attaching conjunctival autografts in pterygium surgery: a systematic review with meta-analysis and trial sequential analysis of evidence
Source: Oncotarget. 2017 Apr 18;8(25):41487–97. doi: 10.18632/oncotarget.17195 (PMC5522296; doi:10.18632/oncotarget.17195)
Supplement: Supplementary file 1 [file oncotarget-08-41487-s001.pdf]

# Efficacy of fibrin glue versus sutures for attaching conjunctival autografts in pterygium surgery: a systematic review with meta-analysis and trial sequential analysis of evidence

## Supplementary Materials

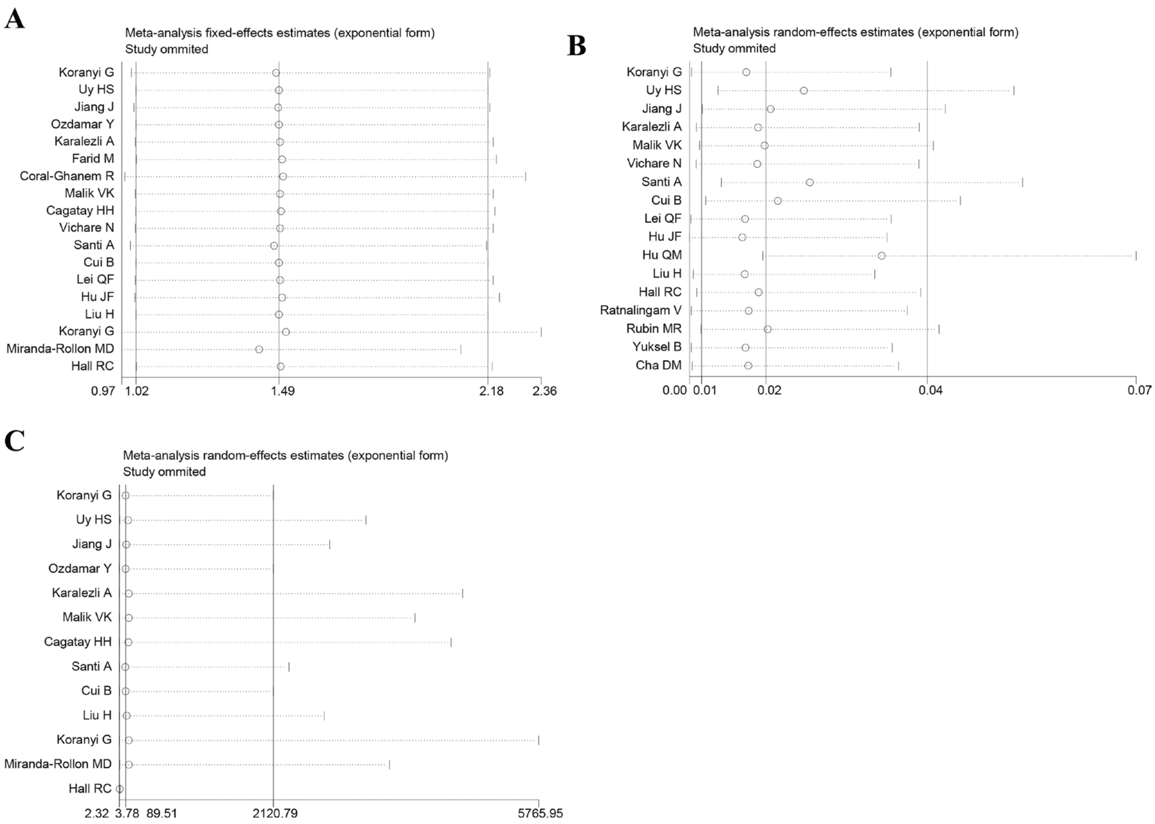


**Supplementary Figure 1:** (**A**) The sensitivity analysis in the recurrence rate group. (**B**) The sensitivity analysis in the surgical duration group. (**C**) The sensitivity analysis in the complication rate group.

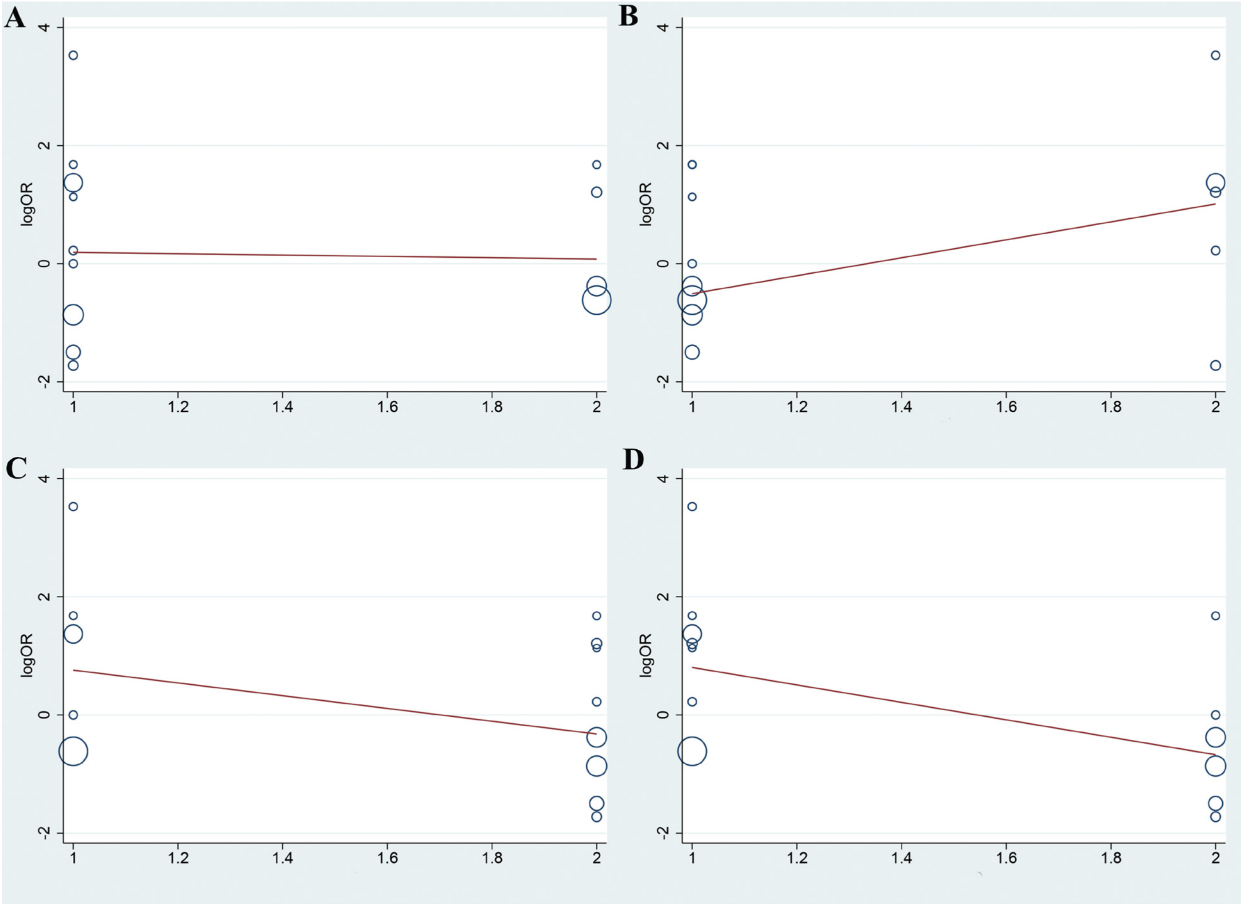


**Supplementary Figure 2: Meta-regression in the recurrence rate group.** (**A**) region, (**B**) sample size, (**C**) suture material, (**D**) follow up duration.

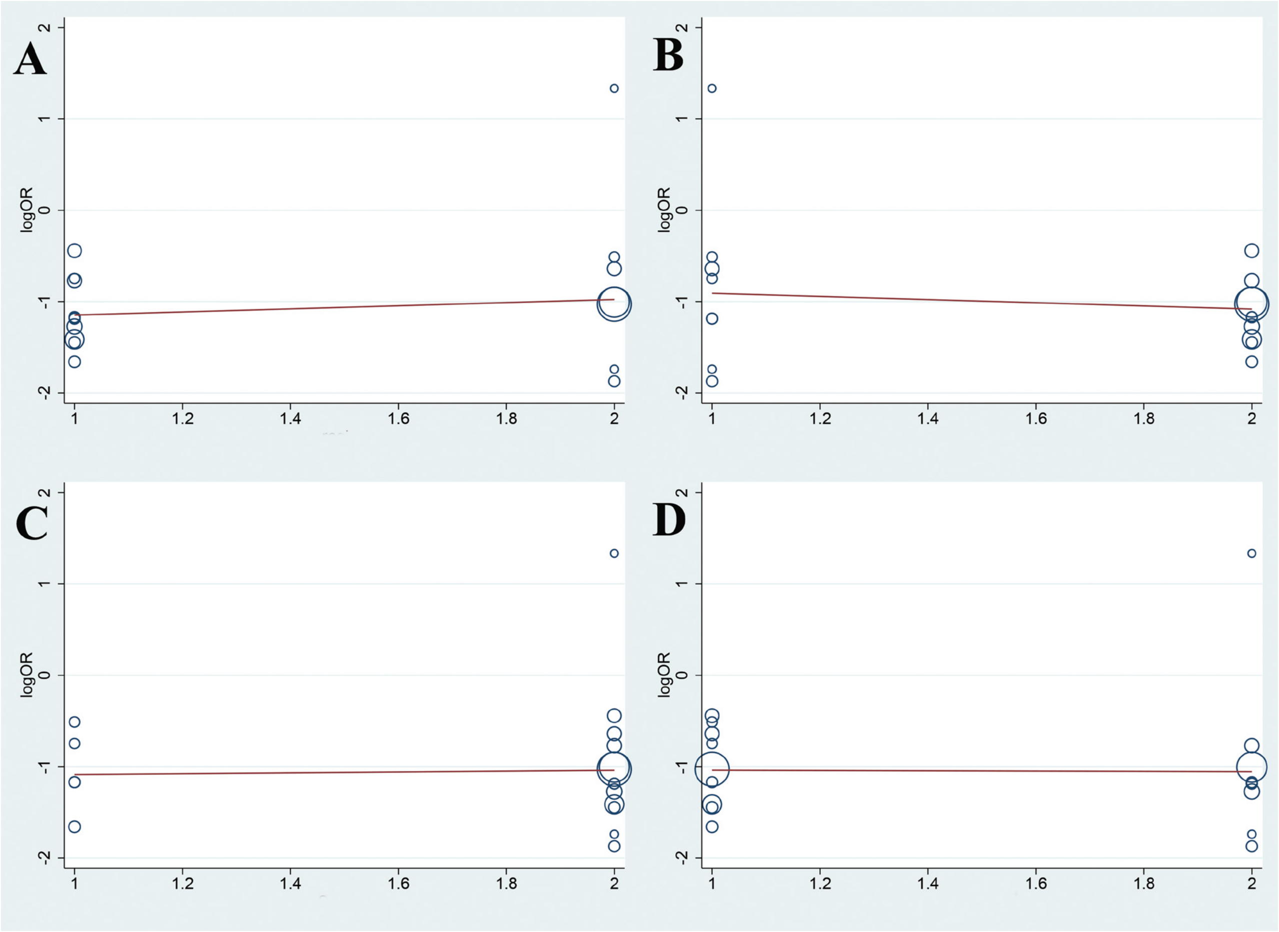


**Supplementary Figure 3: Meta-regression in the complication rate group.** (**A**) region, (**B**) sample size, (**C**) suture material, (**D**) follow up duration.

## Supplementary Table 1: Results of meta-regression analyses

| | recurrence rate group | | complication rate group | |
|---|---|---|---|---|
| | ***P* value** | **95% CI** | ***P* value** | **95% CI** |
| Region | 0.671 | (0.514–2.73) | 0.895 | (0.142–5.619) |
| Sample size | 0.727 | (0.301–2.353) | 0.035 | (1.140–18.296) |
| Suture material | 0.937 | (0.309–3.549) | 0.182 | (0.639–1.803) |
| Follow-up | 0.967 | (0.429–2.252) | 0.057 | (0.497–1.050) |
